# Exosomal encapsulation of miR-3198 promotes proliferation and migration of trophoblasts in preeclampsia

**DOI:** 10.1007/s10815-024-03104-x

**Published:** 2024-03-27

**Authors:** Yuchen Li, Yanling Yu, Dejun Li, Lei Li

**Affiliations:** 1 Department of Obstetrics and Gynecology, Shandong Provincial Hospital Affiliated to Shandong First Medical University, Jinan, 250021 Shandong China; 2 The Laboratory of Medical Science and Technology Innovation Center (Institute of Translational Medicine), Shandong First Medical University (Shandong Academy of Medical Sciences) of China, Jinan, 250021 Shandong China; 3 Department of Obstetrics and Gynecology, Shandong Provincial Hospital, Cheeloo College of Medicine, Shandong University, Jinan, 250021 Shandong China; 4 Department of Obstetrics and Gynecology, People’s Hospital of Xiajin County, Dezhou, 253299 Shandong China; 5 Department of ICU, Shandong Provincial Hospital Affiliated to Shandong First Medical University, Jinan, Shandong China

**Keywords:** Preeclampsia, miRNA, Exosomes, Trophoblasts

## Abstract

**Purpose:**

Preeclampsia (PE) is a vascular remodeling disorder cloesly linked to trophoblast dysfunction, involving defects in their proliferation, migration, and apoptosis. Maternal exosomal microRNAs (miRNAs) have been reported to play pivotal roles in the development of PE. However, the mechanism underlying the role of maternal exosomes in trophoblast dysfunction regarding the development of PE is poorly understood.

**Methods:**

Plasma exosomes from maternal peripheral blood were collected from pregnant women with PE and from those with normal pregnancy. Bioinformatics analysis was used to identify significantly differentially expressed miRNAs under these two conditions. The expression of the miR-3198 gene in plasma exosomes was detected using quantitative real-time polymerase chain reaction. Dual luciferase reporter assay was used to confirm binding of miR-3198 and 3′UTR region of WNT3. Cell proliferation was examined using the Cell Count Kit-8 and EdU assays, and flow cytometry was performed to detect apoptosis and cell cycle. Changes in cell migration were examined using transwell and scratch assays.

**Results:**

Patients with PE showed decreased expression of plasma-derived exosomal miR-3198. The proliferation and migration abilities of HTR-8/SVneo and primary human trophoblast cells were both improved when cocultured with miR-3198-rich exosomes. Exposure to miR-3198-enriched exosomes facilitated cell cycle progression but reduced apoptosis in HTR-8/SVneo cells. Notably, overexpression of miR-3198 partially prevented the inhibitory effects of WNT3 on proliferation and migration in HTR-8/SVneo cells.

**Conclusion:**

Exosomal miR-3198 in the maternal peripheral blood may regulate the biological functions of trophoblasts by targeting WNT3 and influence the development of diseases of placental origin.

**Graphical Abstract:**

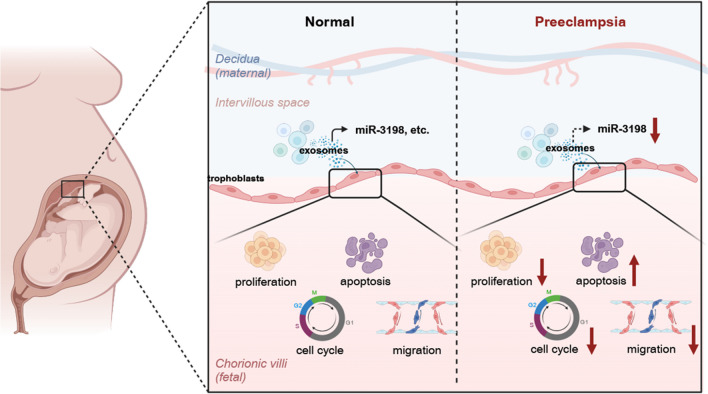

## Introduction

Preeclampsia (PE) is a key cause of maternal and fetal perinatal morbidity and mortality worldwide, affecting 3–5% of all pregnancies [1]. Although maternal inflammation and endothelial dysfunction may be related to the development of PE, the etiology of PE is poorly understood [2, 3]. The failure of uterine vascular remodeling is a widely known mechanism of PE, with extravillous trophoblast invasion playing an crucial role in this process. Several studies have revealed different microRNA (miRNA) profiles in the placenta and peripheral circulation of patients with PE.

miRNAs are noncoding RNAs regulating approximately 90% of human genes, generally by binding to the 3′-untranslated region (UTR) of the mature target messenger RNAs (mRNAs) and leading to their degradation. miRNAs are vital genomic factors in development and physiology. Increasing evidence has shown that miRNAs play a pivotal role in the regulation of trophoblast proliferation, invasion [4–7], epithelial–mesenchymal transition (EMT) [8], and ferroptosis [9]. miR-3198 has been implicated in many diseases involved in multisystem diseases, such as nasopharyngeal carcinoma [10], ovarian cancer [11], aortic aneurysm [12], and periodontitis [13]. However, the role of miR-3198 in the development of PE remains poorly understood.

Exosomes are extracellular vesicles (EVs) with a diameter of approximately 30–100 nm. EVs are organized vesicles that originate from endocytic processes and are released constitutively or in response to stimulus through multivesicular bodies during exocytosis [14]. Exosomes are commonly used as “endogenous vectors,” capable of delivering cargo to regulate nearby cells in a paracrine manner [15]. Exosomes can also exert a systematic effect through blood transport. The exosomal cargo includes many types of molecules, such as miRNA, DNA, and proteins [16]. The contents of exosomes in PE differ from those in normal pregnancy, indicating that exosomes can be used as biomarkers for PE diagnosis [17].

During pregnancy, many endogenous and exogenous factors control pathophysiological changes. However, maternal–fetal communication provides insights into pregnancies with complications. In this study, we investigated the function of miR-3198 in mediating the proliferation and migration of trophoblasts and its role in maternal–fetal communication via exosomes.

## Materials and methods

### Patient samples

Patients, aged 18–32 years, were recruited at the Department of Obstetrics, Shandong Provincial Hospital, affiliated with Shandong First Medical University (Jinan, China). Written informed consent was obtained from all participants. PE was defined according to the guidelines of the American College of Obstetricians and Gynecologists. Serum samples were prepared from blood collected from patients with PE (*n* = 10) and normal pregnancy (controls; *n* = 10).

Inclusion criteria were as follows: new-onset hypertension systolic blood pressure at least twice ≥ 140 mmHg and/or diastolic blood pressure ≥ 90 mmHg and proteinuria (≥ 300 mg), after 20 weeks of gestation. The exclusion criteria were as follows: (i) multiple pregnancies, (ii) smoking, and (iii) history of other clinical disorders, such as chronic hypertension or renal disease. More clinical information about the sample is found in Table 1. Peripheral blood (PB) samples were collected from patients with PE in EDTA-anticoagulant tubes (5 mL per sample) and centrifuged at 2000 × g for 15 min at 4 °C within 1 h to separate the plasma and blood cells. The plasma samples were immediately stored at − 80 °C for further use. This study complied with the World Medical Association Declaration of Helsinki guidelines and was approved by the Ethics Committee of the Shandong Provincial Hospital Affiliated to Shandong First Medical University (SWYX:NO.2023–279).

**Table 1:** The clinical characteristics of the study population

Characteristics	Normal (*n* = 10)	Preeclampsia (*n* = 10)
Maternal age (years)	30.90 ± 3.90	33.90 ± 2.42
Gestational age (weeks)	39.14 ± 0.38	36.92 ± 1.02****
Systolic pressure (mmHg)	106.40 ± 9.40	159.80 ± 13.69****
Diastolic pressure (mmHg)	71.90 ± 10.40	101.7 ± 8.34****
Birth weight (g)	3391.00 ± 400.80	2405.00 ± 626.10***
Proteinuria	Negative	Positive

Data are shown as means ± SDs

All results are reported after adjustment for baseline values using the unpaired *t*-test

***p<0.001, ****p<0.0001

### Primary human trophoblast cell isolation

Primary human trophoblast cells were isolated as previously described [18, 19]. The villus tissue of maternal surface was taken, and blood clots and fetal 16 membranes were removed. After washing three times with sterile D-Hanks, the placentas were cut into pieces of about 1 mm^3^. A mixture of 0.25% trypsin, 0.1% collagenase, and deionized water was added to the tube and incubated for digestion in a 37 °C water bath for 25 min. When the single cell formation was observed under a microscope, the supernatant was collected, filtered, and centrifuged at 250 × g for 10 min. The resultant cell pellet was resuspended in Dulbecco’s modified Eagle’s medium (DMEM)/Ham’s F12 (1:1) suspension supplemented with 10% fetal bovine plasma (FBS). Cells were maintained in DMEM (Gibco, NY, USA), supplemented with 10% FBS, 25 mM 17 HEPES, 2 mM glutamine, 100 g/mL streptomycin, and 100 UI/mL penicillin, and incubated in humidified air with 5% CO_2_ at 37 °C.

### Cell culture and transfection

HTR-8/SVneo cells were maintained in RPMI-1640 (Gibco, NY, USA), supplemented with 10% fetal bovine serum (FBS), at 37 °C in a humidified incubator with 5% CO2. HEK293T cells were cultured in high-glucose Dulbecco’s modified Eagle’s medium (Gibco) supplemented with 10% exosome-free FBS at 37 °C in a humidified incubator with 5% CO2.

HTR-8/SVneo cells in the logarithmic phase of culture were seeded in a six-well plate at 3 × 105 cells/well and cultured to 60% confluence. The cells were transfected with 50 nM miR-3198 mimic or miR-NC (Ribobio Co., Ltd., Shanghai, China) using Lipofectamine 2000 reagent (11668019, Invitrogen, CA, USA) per the manufacturer’s instructions. After transfection for 48 h, the HTR-8/SVneo cells were harvested and used for functional assays.

### Exosome isolation

Exosomes were separated from PB plasma following a previously described protocol with modifications [20, 21]. Briefly, PB plasma was mixed with an equal volume of cold phosphate-buffered saline (PBS), and the mixture was sequentially centrifuged at 300 × *g* for 10 min, at 2000 × *g* for 30 min, and at 12,000 × *g* for 45 min. After centrifugation at 2000 × *g*, the supernatant was gently filtered through a 0.22-µm sterile filter (Steritop, Millipore, USA) and centrifuged twice at 120,000 × *g* for 70 min (Beckman Coulter). The supernatant was removed, and the pellets were resuspended in 200 µL sterile PBS. All the centrifugation steps were performed at 4 °C. The protein concentration of exosomes was measured using the bicinchoninic acid method. The resuspended exosomes were immediately frozen at − 80 °C or used in subsequent experiments.

### Electroporation of exosomes

The electroporation method has higher purity and stability, and it can directly load biologically active substances such as drugs and siRNAs into exosomes without needing other media [22]. Electroporation uses a pulsed electric field to apply voltage to exosomes, resulting in the opening of the exosome membrane and allowing drugs to enter the exosomes. Electroporation requires the collection of exosomes from the culture medium, followed by purification of the obtained exosomes through ultracentrifugation or other methods. The transfection complex is prepared by packaging the exosomes with DNA or RNA. Finally, electroporation parameters are adjusted to ensure optimal transfection efficiency. Parameters such as voltage, pulse duration, and electrode spacing need to be optimized according to cell type and exosome type [23–25]. Exosomes and the miR-3198 mimic were placed on ice for 10 min. The exosomes were diluted to 0.5 mg/mL in electroporation buffer. The miR-3198 mimic was added at a final concentration of 100 nM. The mixture was transferred to a precooled 2 mm cuvette and subjected to three 400 V, 125 µF capacitance pulses for 5 ms. After electroporation, the exosomes were placed on ice for 10 min.

### Coculture of exosomes with HTR-8/SVneo cells

HTR-8/SVneo cells were divided into a blank group (HTR-8/SVneo cells cultured under normal conditions), an NC-EXO group (exosomes electroporated with miR-NC and cocultured with HTR-8/SVneo cells), and an miR-3198-EXO group (exosomes electroporated with the miR-3198 mimic and cocultured with HTR-8/SVneo cells). HTR-8/SVneo cells were cocultured with electroporated exosomes in accordance with the grouping in 100 µg/mL for 48 h and used for further studies.

### Western blot

The Western blotting was carried out as our previous protocol [21]. All proteins were extracted using RIPA-PMSF methods and quantified through BCA kit (Solarbio, Beijing, China). Briefly, 20 μg of loaded protein was separated with 7.5%, 10%, or 12.5% SDS‒PAGE (Epizyme, Shanghai, China) and then transferred onto PVDF membranes (Millipore, MA, USA). The membranes were blocked with 5% nonfat milk at room temperature for 1 h and incubated at 4 °C overnight with the primary antibodies. The next day, the membranes were incubated with the corresponding secondary antibody (Proteintech) for 2 h. Finally, the blots were visualized by Amersham Imager 600 system (GE, Boston, MA, USA). Primary antibodies were listed as follows: CD63 (1:1000, ab134045, Abcam), TSG101 (1:1000, ab125011, Abcam), CD81 (1:1000, ab79559, Abcam), WNT3 (1:1000, ab116222, Abcam), and GAPDH (1:3000, ab9485, Abcam).

### RNA extraction and quantitative real-time polymerase chain reaction (qRT-PCR)

Total exosomal RNA of PB plasma from patients with PE or controls was extracted using the AG RNAex Pro reagent (Accurate Biotechnology, Jinan, China) according to the manufacturer’s instructions. The concentration of total RNA was determined using a NanoDrop2000 spectrophotometer (Thermo Fisher Scientific). To avoid RNA degradation, it was reverse transcribed into cDNA as soon as possible. For reverse transcription into cDNA, RNA was treated with the Evo M-MLV RT Kit with gDNA Clean for qPCR (Accurate Biotechnology). The cDNA was used for qRT-PCR with SYBR Green Premix Pro Taq HS qPCR Kit (Accurate Biotechnology), and the level of expression of genes relative to GAPDH was determined using the 2^−△△CT^ method [14]. All the primers were synthesized by JEKAIYER (Shandong, China). The primer sequences are shown in Table 2.

**Table 2:** Sequences of primers used for qRT-PCR

Name		Primers for qRT-PCR (5′-3′)
U6	Forward	GTGCTCGCTTCGGCAGCACATAT
	Reverse	AGTGCAGGGTCCGAGGTATT
miR-3198	Forward	CGGTGGAGTCCTGGGGAA
	Reverse	AGTGCAGGGTCCGAGGTATT
miR-11400	Forward	CGCGTCGGCTGTGTATCTC
	Reverse	AGTGCAGGGTCCGAGGTATT
miR-376b-3p	Forward	GCGCGATCATAGAGGAAAATC
	Reverse	AGTGCAGGGTCCGAGGTATT

*qRT-PCR* quantitative real-time polymerase chain reaction

### Cell counting kit-8 (CCK-8) assay

HTR-8/SVneo cells subjected to pretreatment (transfection or coculture with exosomes) were incubated along with 10% CCK-8 (Bioss, Beijing, China) solution in 96-well plates for 2 h. Thereafter, the absorbance at 450 nm was measured using a microplate reader, according to the manufacturer’s instructions.

### Migration assay

HTR-8/SVneo cells were resuspended in a plasma-free medium and plated in the upper chamber of a transwell plate (Corning, NY, USA). Completed medium containing 10% FBS was added to the lower chamber. After incubation for 24 h, non-migrating cells were removed using a cotton swab, and migrating cells were stained with 0.1% crystal violet. In each well, three microscopic fields were photographed randomly, and the number of cells was counted manually.

### Scratch assay

To determine the migration ability of HTR-8/SVneo cells, pretreated cells were seeded in six-well plates and incubated until they reached a confluent state. The cell monolayer was scratched with a 200-μL pipette tip, and the wells were rinsed with PBS to remove detached cells. After culturing in plasma-free medium for 24 h, images were captured at 0 and 24 h. The width of the scratches was measured using the ImageJ software (NIH, USA).

### Apoptosis assay

HTR-8/SVneo cells were cultured in the presence of exosomes for 24 h. The apoptosis assay was performed using an apoptosis kit (BD Biosciences) according to the manufacturer’s instructions. Briefly, cells were resuspended at a concentration of 1 × 10^6^ cells/mL and 500 μL binding buffer was mixed with 100 μL cell suspension. The staining solution was added and gently blended on ice for 15 min in the dark. Flow cytometry (BD Accuri™ C6 Plus) was used to detect the apoptotic cells within 1 h.

### Cell cycle assay

Cell cycle assay was performed using a cell cycle detection kit (Key GEN BioTECH, Jiangsu, China) according to the manufacturer’s instructions. Briefly, harvested HTR-8/SVneo cells were rinsed with PBS and then fixed with precooled 70% ethanol at − 20 °C. After overnight incubation, the cells were rinsed with PBS twice. RNase A and propidium iodide (PI) were mixed at a 1:9 ratio. After removing the supernatant, 500 μL PI/RNase A staining solution was added to the cells, and the cells were incubated at room temperature for 40 min. Flow cytometry (BD Accuri™ C6 Plus) was performed immediately to analyze the cell cycle.

### Dual-luciferase reporter assay

The seed sequence in the 3-UTR of human WNT3 mRNA binding by miR-3198 was analyzed by TargetScan. 3-UTR of WNT3 and a sequence with mutant nucleotides in the miR-3198 binding site were inserted into pGL3 vector for luciferase. HEK393t cells were co-transfected with luciferase vector and miR-3198 mimics or control oligonucleotides. Luciferase activity was examined using the Dual-luciferase Reporter Assay System in accordance with the manufacturer’s protocol (Promega, USA) at 48 h after transfection.

### Statistical analysis

Data were analyzed using the GraphPad Prism software version 9.0. Data were compared using analysis of variance (ANOVA) or *t*-test. A value of *p* < 0.05 was considered to indicate statistical significance.

## Results

### Exosomal miRNA sequencing and bioinformatics analysis

**Fig. 1:**
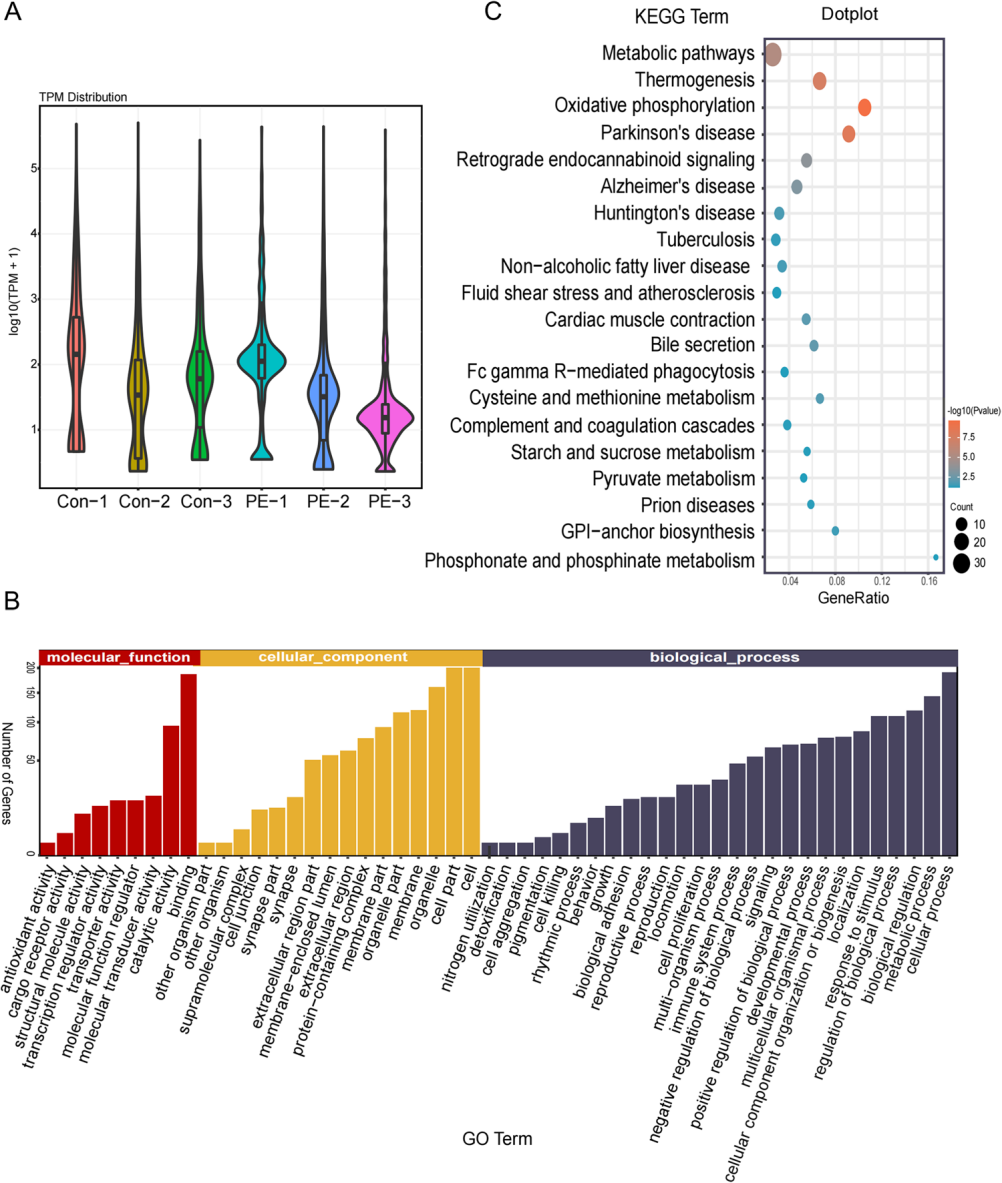
Bioinformatic analysis of miRNAs. miRNA sequencing was performed to detect differential expression of exosomal miRNAs in the plasma of women with PE and normal pregnancy (*n* = 3). **A** Violin plot showing expression of miRNAs in each sample. *X*-axis indicates the different samples, *Y*-axis shows the quantity of miRNAs under related expression levels using log10(TPM + 1), and the width of violin indicates the number of miRNAs corresponding to a particular expression level. **B** GO enrichment analysis of target genes represented using the bar plot. The length of bars indicates the number of genes enriched with a specific term. **C** KEGG enrichment analysis of target genes represented using the dot plot. Gene ratio indicates enriched genes divided by the total number of genes annotated with a specific KEGG term. Smaller *p* values refer to significant differences, and the diameter of the dot represents the counts of genes enriched in the specific KEGG pathway. TPM = *T* × 10^6^/*N* (*T* refers to the tag of a particular miRNA; *N* refers to the total tags of miRNAs. PE, preeclampsia; TPM, tags per million; GO, gene ontology; KEGG, Kyoto encyclopedia of genes and genomes

To investigate the potential differences in exosomal miRNAs in plasma between PE and normal pregnancy, we performed exosomal miRNA sequencing. Statistical significance was set at *p* < 0.05. The miRNA expression levels in the control and PE groups are shown in Fig. 1A. Target genes of the differentially expressed miRNAs were predicted using RNAhybrid, miRanda, and TargetScan. Gene ontology (GO) enrichment analysis was performed to determine the target genes enriched in molecular function, cellular components, or biological processes (Fig. 1B). To further identify the biological pathways involved, we performed Kyoto encyclopedia of genes and genomes (KEGG) enrichment analysis for the predicted target genes. The top 20 significantly enriched pathways are shown in Fig. 1C, including metabolic pathways, thermogenesis, and oxidative phosphorylation.

Based on bioinformatics analysis, we selected miR-3198, miR-376b-3p, and miR-11400 as candidate miRNAs for further investigation of their roles in PE.

### Downregulation of miR-3198-exo derived from plasma in PE

**Fig. 2:**
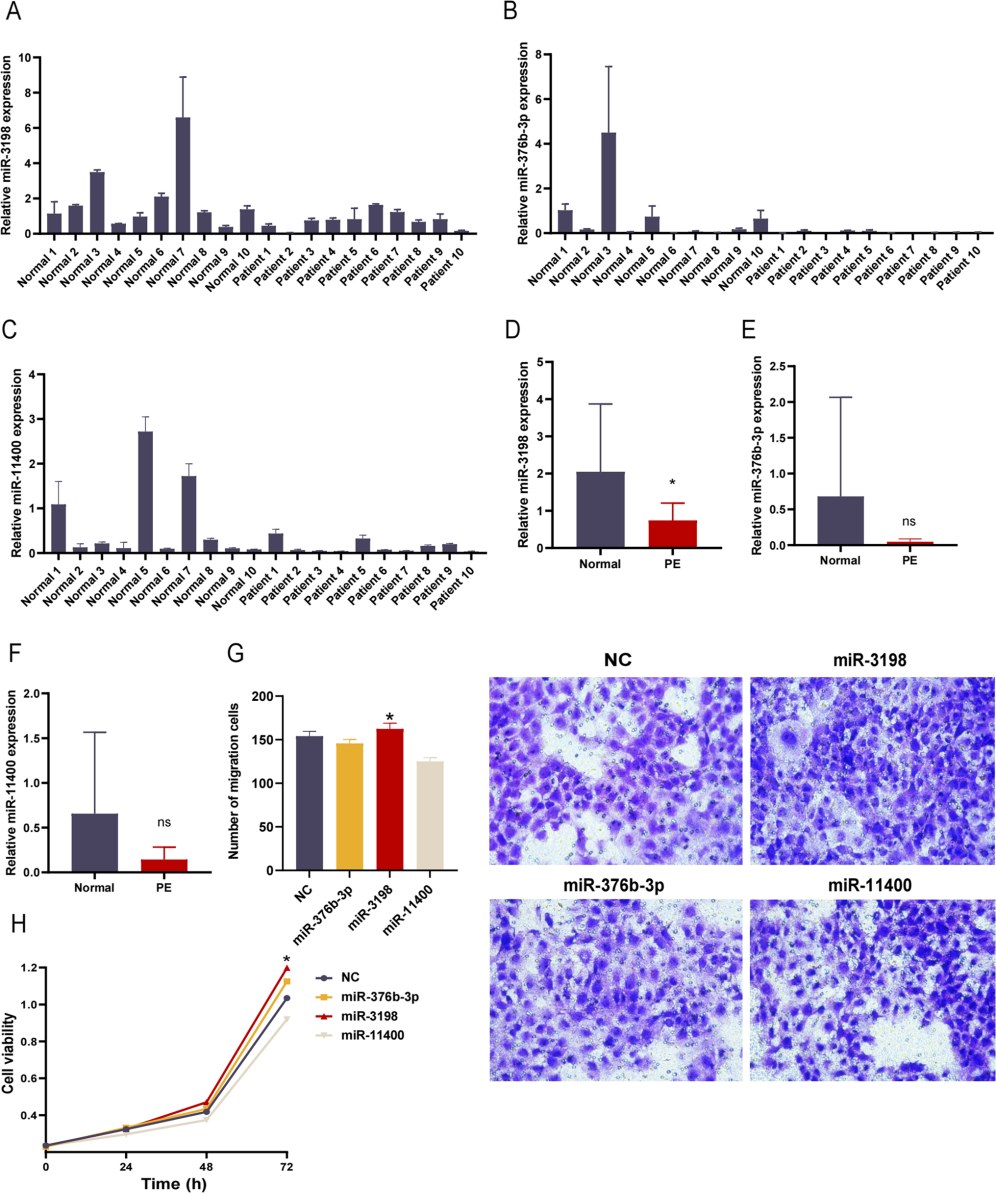
Identification of the imbalance in expression of exosomal miR-3198 and adverse effects of miR-3198 on HTR-8/SVneo cells. **A**–**C** Expression levels of miR-3198, miR-376b-3p, and miR-11400 in plasma-derived exosomes from women with PE and normal pregnancy (*n* = 8). **D**–**F** Statistical analysis for the expression levels of the three miRNAs in the normal and PE groups. HTR-8/SVneo cells were transfected with the indicated miRNAs and negative control separately (50 nM) for 48 h. **G** Cell migration analyzed using the transwell assay (*n* = 3). **H** Cell viability analyzed using the CCK-8 assay (*n* = 3). *p < 0.05. PE, preeclampsia; CCK-8, cell counting kit-8; ns, no significance

To validate the sequencing results, we determined the expression of miRNAs in serous exosomes using qPCR. Unexpectedly, the expression of exosome-derived miR-3198 in PE was significantly lower than that in normal pregnancy (Fig. 2A–F). To explore whether miR-3198, miR-376b-3p, and miR-11400 directly influence trophoblast viability, we transfected the cells with the respective miRNA mimic to improve the levels of related miRNAs and evaluated the cell viability and migration ability. The trophoblast cell line HTR-8/SVneo was selected for subsequent studies. Notably, enhanced miR-3198 expression promoted the viability and migration of HTR-8/SVneo cells, which was consistent with the increased expression in normal clinical samples (Fig. 2H, G). However, miR-376b-3p and miR-11400 mimics did not significantly affect the HTR-8/SVneo cells compared with the corresponding negative control. Therefore, we hypothesized that plasma exosomes participate in the development of PE by disrupting the biological behavior of trophoblasts through the delivery of miR-3198.

### Exosomal exosomal miR-3198 disturbed proliferation, apoptosis, and the cell cycle of HTR-8/SVneo cells

**Fig. 3:**
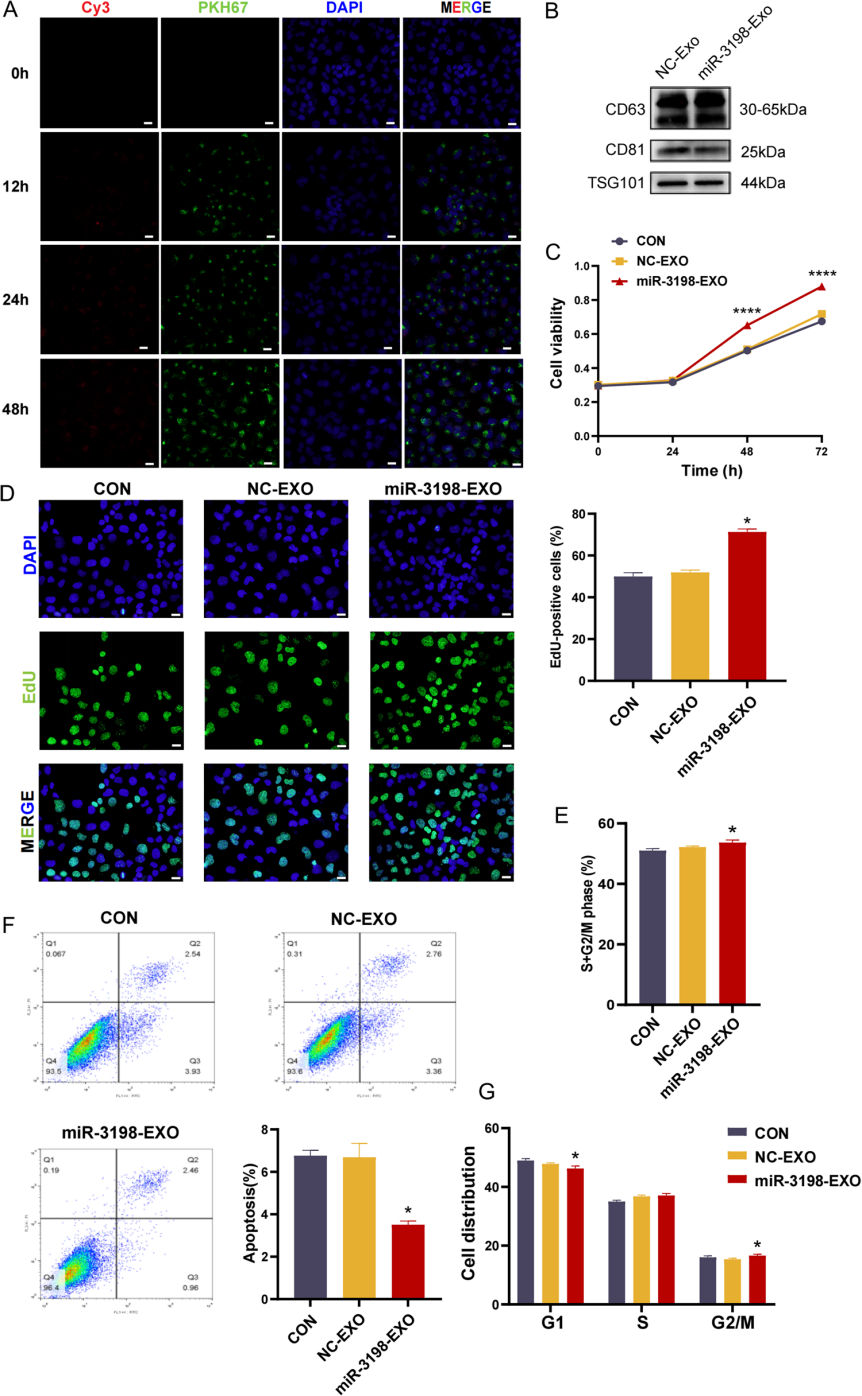
Identification of homeostasis of HTR-8/SVneo cells by exosomal miR-3198. **A** PKH67-labeled exosomes encapsulating Cy3-labeled miRNA mimic were incorporated in HTR-8/SVneo cells. Nuclei are stained with DAPI. Scale bars, 20 µm. HTR-8/SVneo cells were cocultured with different groups of exosomes (100 µg/mL) for 48 h. B Western blot analysis of exosome markers CD63, CD81, and TSG101. **C** Cell viability was determined using the CCK-8 assay (*n* = 3). **D** Cell proliferation was determined using the EdU assay (*n* = 3). DAPI was used to visualize nuclei. Scale bars, 20 µm. Flow cytometry was used to investigate **E**, **G** cell cycle distribution (*n* = 3) and **F** apoptosis of cells by staining with annexin V-FITC and PI (*n* = 3). Scale bars, 20 µm. *p < 0.05; ****p < 0.0001. CCK-8, cell counting kit-8; EdU, 5-ethynyl-2′-deoxyuridine

Based on the direct influence of miR-3198 on trophoblasts, we constructed a physiological in vitro model that cocultured HTR-8/SVneo cells with exosomes loaded miR-3198 via electroporation. This mimics the imbalance in the contents of miR-3198 in exosomes between PE and normal pregnancy. First, the miRNA mimics transported into HTR-8/SVneo cells by exosomes were identified using immunofluorescence (Fig. 3A). Additionally, specific protein markers (CD63, CD81, and TSG101) were used to identify that these vesicles were exosomes (Fig. 3B). We then used CCK-8 and EdU incorporation assays to detect the differences in cell proliferation. Similar to direct transfection, the proliferative activity of HTR-8/SVneo cells was distinctly improved by miR-3198 mimic exosomes (Fig. 3C, D). Next, we used flow cytometry to analyze the cell cycle contribution under miR-3198 mimic exosomes or miR-mimic NC-exosome coculture. Treatment with miR-3198 mimic exosomes promoted the proliferation of HTR-8/SVneo cells in the G2/M and S phases (Fig. 3E). In contrast, the percentage of the miR-3198-EXO group in the Go/G1 phase was lower than that in the NC-EXO group (Fig. 3G). We also investigated whether miR-3198 exosomes intervened in cell apoptosis and found that apoptosis was suppressed in the miR-3198-EXO group, as expected (Fig. 3F). These data demonstrate the effects of exosomal miR-3198 on the promotion of cell proliferation, cell cycle progression, and suppression of apoptosis.

### Exosomal miR-3198 mediated the migration ability of HTR-8/SVneo cells

**Fig. 4:**
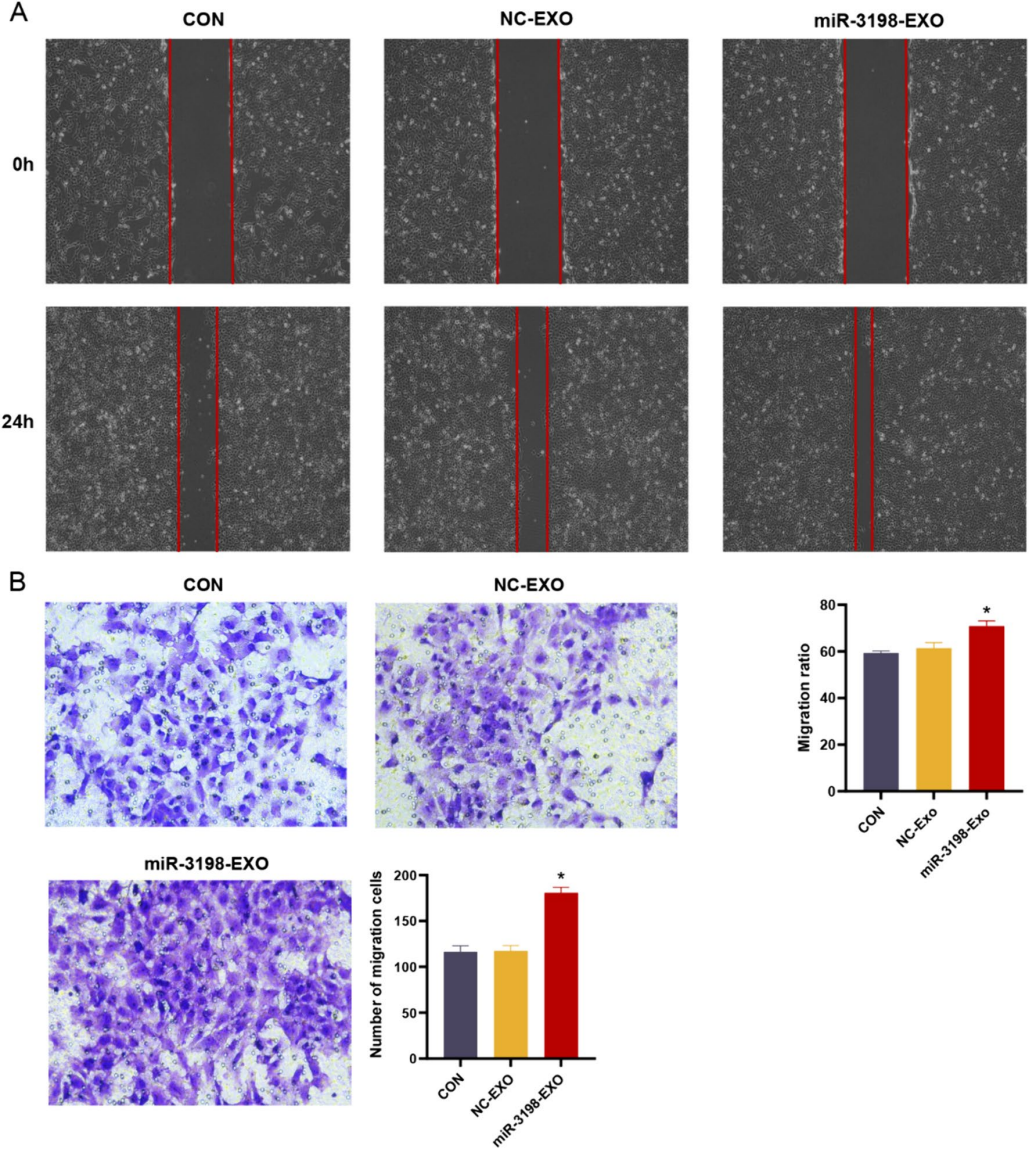
Exosomal miR-3198 enhances the migration ability of HTR-8/SVneo cells. **A** Scratch (*n* = 3) and **B** transwell (*n* = 3) assays were used to determine the cell migration ability. *p < 0.05

We explored whether exosomal miR-3198 affects the migration of trophoblasts, which is tightly associated with vascular reconstruction. The results of scratch and transwell assays indicated enhanced migration ability in the miR-3198-EXO group compared with that in the NC-EXO group (Fig. 4A, B). Briefly, upregulation of exosomal miR-3198 markedly promoted the migration ability of HTR-8/SVneo cells. Thus, exosomal miR-3198 enhanced proliferation and cell cycle progression. In contrast, the rate of apoptosis was suppressed due to the enhanced expression of miR-3198 derived from plasma exosomes.

### Exosomal miR-3198 promoted cell proliferation and migration ability in human primary trophoblast cells

**Fig. 5:**
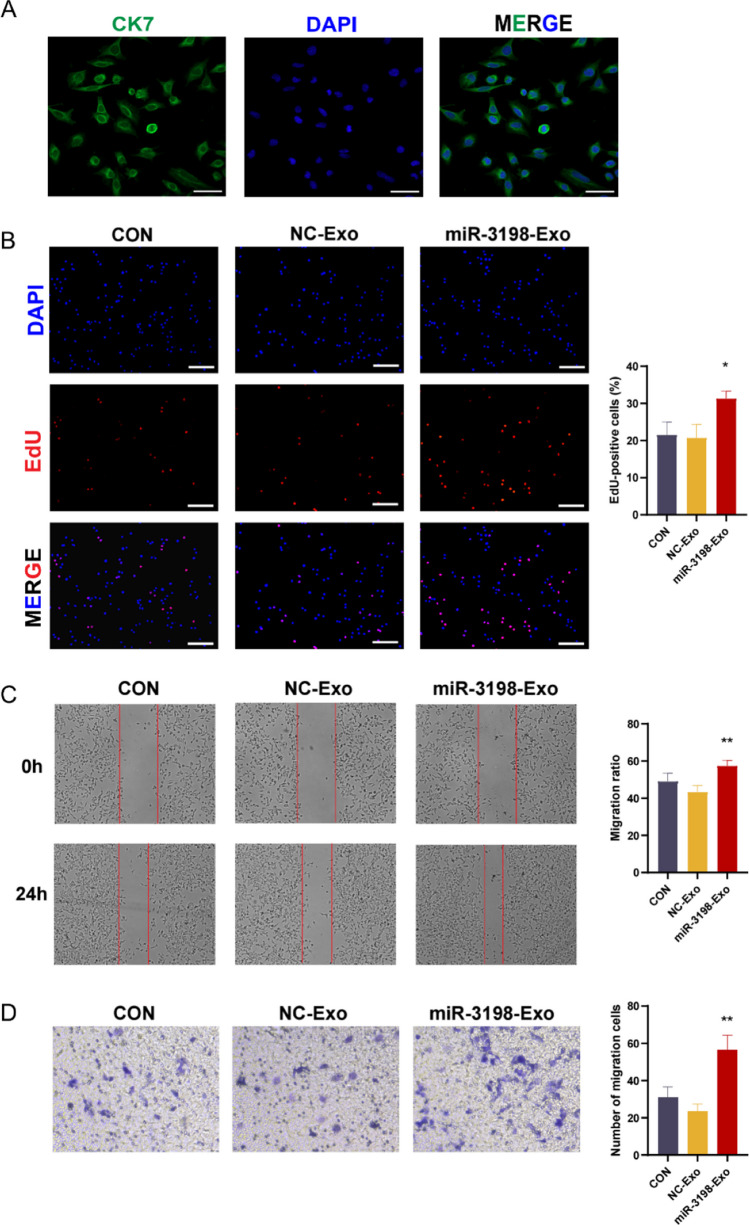
Exosomal miR-3198 enhances the proliferation and migration ability of primary trophoblast cells. **A** Immunofluorescence staining of CK7 for primary trophoblast cell identification. Green fluorescence signals indicate CK7, and the DAPI-stained nuclei are blue. Scale bar, 50 μm. **B** Cell proliferation was determined using the EdU assay (*n* = 3). Hoechst was used to visualize nuclei. Scale bars, 200 µm. **C** Scratch (*n* = 3) and **D** transwell (*n* = 3) assays were used to determine the cell migration ability. *p < 0.05; **p < 0.01

To further identify the functional role of exosomal miR-3198 in human primary trophoblast cell proliferation and migration, human primary trophoblast cells were successfully isolated and identified using immunofluorescence demonstrating that the percentage of CK7-positive cells is over 90% [18, 26] (Fig. 5A). The EdU assay demonstrated that the relative cell proliferation in the miR-3198-EXO group was higher than that in the NC-EXO group (Fig. 5B). Meanwhile, the increased migratory rates were observed in the miR-3198-EXO group in both transwell and scratch assay (Fig. 5C, D). Data revealed that exosomal miR-3198 notably promoted cell proliferation and migration in primary trophoblast cells as compared with the NC-EXO group.

### miR-3198 directly targeted WNT3 in trophoblast cells

**Fig. 6:**
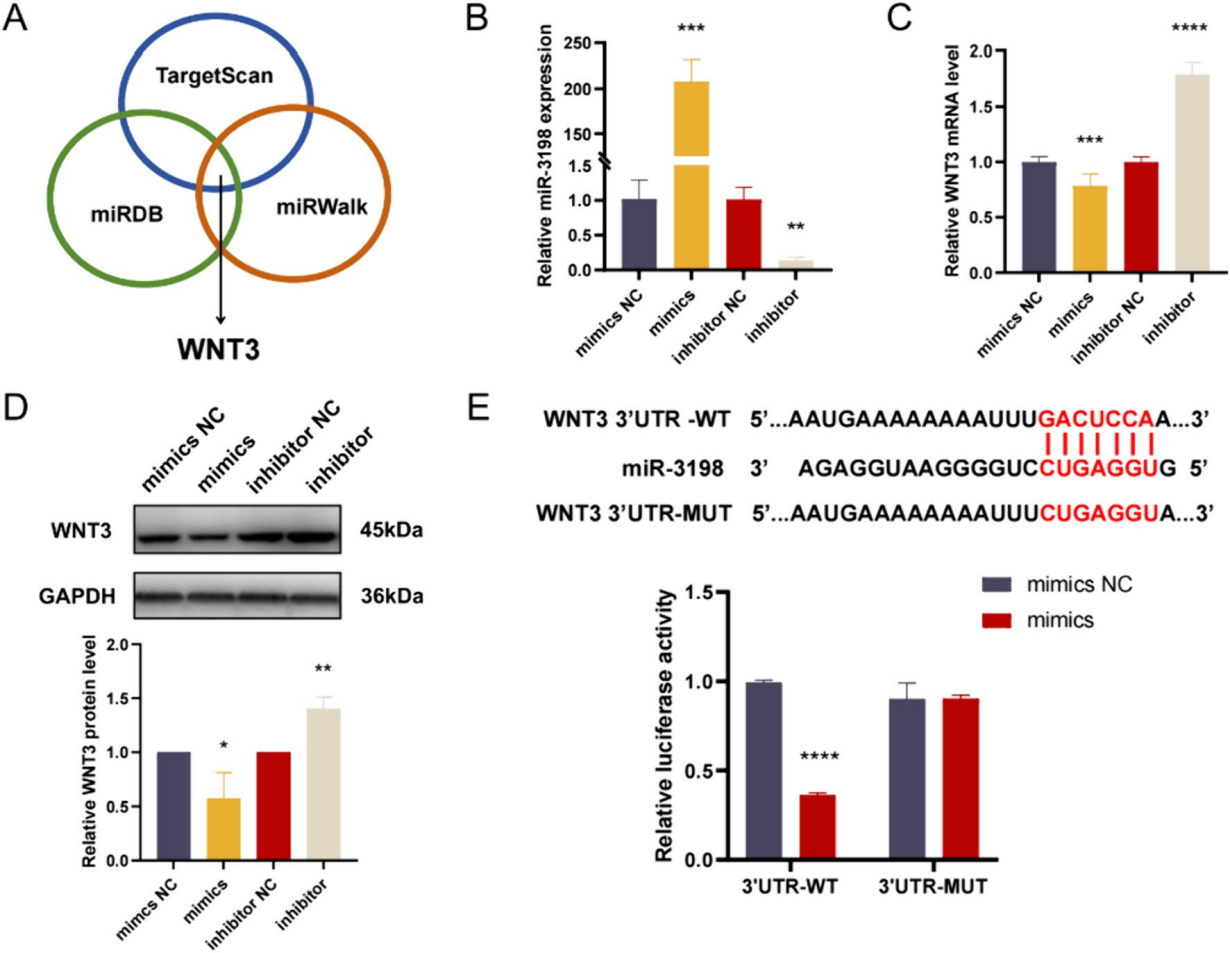
Targeting of WNT3 by miR-3198 in trophoblast cells. **A** Venn diagram analysis showed that WNT3 was among the targeted genes predicted simultaneously in 3 different online databases. **B** Relative expression of miR-3198 in the mimics, inhibitor, and relative NC groups was investigated using qPCR (*n* = 3). **C** Relative expression levels of WNT3 mRNA in the mimic, inhibitor, and relative NC groups respectively (*n* = 3). **D** Relative expression levels of WNT3 protein in the mimic, inhibitor, and relative NC groups respectively (*n* = 3). **E** Regulatory relationship between WNT3 and miR‑3198 was verified using a dual‑luciferase reporter assay (*n* = 3). *p < 0.05; **p < 0.01

The Wnt/β-catenin pathway played a critical role in human placenta and trophoblast development [27], and WNT3 gene was found to be highly expressed in preeclamptic placental tissues [28]. WNT3 was a stronger suggested downstream candidate targeted by miR-3198 (Fig. 6A). To investigate the interactions between miR-3198 and WNT3 in PE, we further examined whether miR-3198 regulated WNT3 in trophoblast cells. Firstly, HTR-8/SVneo cells were transfected with miR-3198 mimics and inhibitor, and we verified the transfection efficiency by qPCR (Fig. 6B). Secondly, analysis of WNT3 mRNA expression was done for HTR-8/SVneo transfected with miR-3198 mimics or miR-3198 inhibitor. In this case, WNT3 mRNA levels were significantly repressed or enhanced following miR-3198 mimics or miR-3198 inhibitor transfection compared to respective negative control groups (Fig. 6C). Finally, we used Western blot assay to study WNT3 protein expression in HTR-8/SVneo miR-3198 mimics and inhibitor transfected cells. It was found that WNT3 protein expression was decreased in the miR‑3198 mimics group and increased in the miR‑3198 inhibitor group (Fig. 6D). As shown in Fig. 6E, miR-3198 was predicted to target 3′UTR of WNT3 transcript by bioinformatic assay; moreover, further luciferase assays evidenced that miR-3198 directly targeted 3′UTR of WNT3 transcript. Therefore, it is concluded that exosomal miR-3198 directly targeted WNT3 in trophoblast cells.

### miR-3198 positively regulates the proliferation and migration of HTR8/SVneo cells by targeting WNT3

**Fig. 7:**
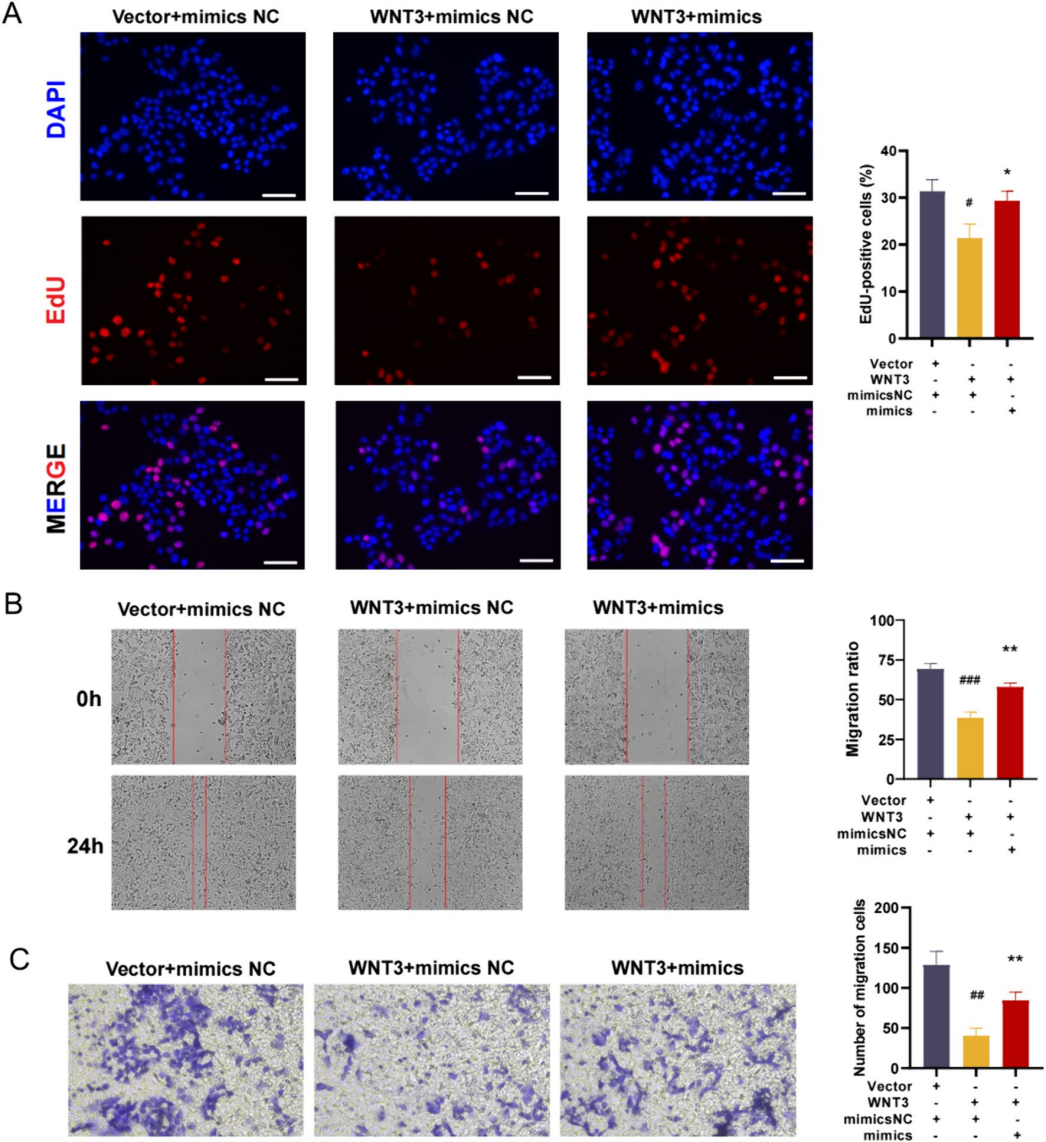
miR-3198 promoted HTR8/SVneo cell proliferation and migration by targeting WNT3. **A** Cell proliferation in trophoblast cells under transfection of miR-3198 and WNT3-OE plasmid (*n* = 3). Scale bar, 100 μm. **B** Scratch (*n* = 3) and **C** transwell (*n* = 3) assays were used to determine the cell migration ability under transfection of miR-3198 and WNT3-OE plasmid. *p < 0.05; **p < 0.01

Transfection of HTR-8/SVneo cells with pcDNA-WNT3 overexpression (OE) or combination of miR-3198 mimics and WNT3 OE plasmid was done to clarify the effects of miR-3198 and WNT3 in PE. EdU assay showed decreased growth in trophoblast cells transfected with WNT3 OE but significantly alleviated when co-transfected with miR-3198 mimics and WNT3 OE plasmid (Fig. 7A). Consistently, transwell and scratch assays showed an inclined cell migration in trophoblast cells transfected using WNT3 OE but a significant increase in trophoblast cells co-transfected with miR-3198 mimics and pcDNA-WNT3 plasmid (Fig. 7B, C). Overall our observations indicate that the protective impacts of exosomal miR-3198 derived from plasma on trophoblast cells by targeting WNT3 to alleviate the cell proliferation and cell migration.

## Discussion

Preeclampsia is a pregnancy complication that threatens maternal and fetal safety. Successful establishment of vascular communication in the placenta mainly depends on the remodeling of the maternal spiral artery, which is related to trophoblast behavior. Inadequate reconstruction of the spiral artery is one of the most crucial events in the occurrence and development of PE. Despite studies on the multiple underlying mechanisms, the pathogenesis of PE is poorly understood. Exosomes encapsulating cargo are involved in substance exchange and communication. Noncoding RNAs encapsulated in exosomes participate in several physiological and pathological processes during pregnancy, such as PE [29–32], gestational diabetes mellitus [20, 33], recurrent spontaneous abortion [34–36], and preterm labor [37–39]. Different profiles of plasma exosomal miRNAs have been investigated in healthy and PE pregnancies [40, 41]. Recently, exosomal miRNAs have been detected in both the placenta and maternal plasma of patients with PE to determine the pathologies of maternal or placental origin in PE [42]. Because of the essential correlation between exosomal cargo and PE, we gained insights into the diversity of plasma exosomal miRNAs and demonstrated that exosomes encapsulating miR-3198 disturb the biological functions of trophoblasts, providing a novel explanation for the development of PE.

In our study, differences in exosomes derived from the PB plasma of women with PE and healthy pregnancies were analyzed via miRNA sequencing and bioinformatics techniques. Exosomal miR-3198 showed a statistically significant difference and drew our attention for further investigation. To further confirm the bioinformatics results, we used qPCR to detect exosomal miR-3198 in clinical specimens. Exosomes enriched with miR-3198 were successfully constructed using electroporation and cocultured HTR-8/SVneo cells, and their effect on proliferation, apoptosis, cell cycle, and migration was determined. Similar results were obtained in primary human trophoblast cells. Subsequently, we found that miR-3198 could significantly improve the proliferation and migration of HTR8/SVneo cells by targeting WNT3 mRNA. Similar investigations have provided similar conclusions; a previous study reported that the WNT3 gene was found to be highly expressed in preeclamptic placental tissues [28]. Combined with our research findings, our studies may give possible upstream molecular mechanism of WNT3. Overall, our study demonstrates that the upregulation of miR-3198 encapsulated in exosomes promotes trophoblast proliferation, cell cycle progression, and migration. However, the overexpression of exosomal miR-3198 inhibited apoptosis in vitro. These results suggest that exosomal miR-3198 deficiency may play an important role in the mechanism of PE and is worthy of intensive discussion.

In a previous study, the involvement of miR-3198 in several diseases was investigated. In recurrent epithelial ovarian cancer (EOC), significant overexpression of miR-3198 compared with that in primary EOC was reported to be a potential biomarker for EOC recurrence [11]. Decreased miR-3198 expression is associated with the pathogenesis of aortic aneurysm [12]. In human periodontal ligament cells, miR-3198 downregulates osteoprotegerin expression under mechanical stress to stimulate osteoclastogenesis [43]. Aberrant expression of miR-3198 in gingival crevicular fluid is related to the presence and severity of periodontitis [13]. Breast cancer cells treated with usnic acid showed increased expression of apoptosis-related genes and promotion of programmed cell death, accompanied by decreased levels of miR-3198 [44]. Maternal plasma EVs have been characterized by the concentration and changeable miRNA content during non-pregnancy, healthy pregnancy, and gestational vascular complications, including gestational hypertension and PE. The lowest levels of hsa-miR-16-5p and hsa-miR-210 were detected in EVs from PE, with the highest levels in healthy pregnancy, indicating their involvement in maternal pathophysiology [42].

Our findings suggest that miR-3198 shuttling by maternal plasma exosomes is positively correlated with cellular proliferation, migration, and progression of the cell cycle and negatively correlated with cellular apoptosis. In addition, deficiency of exosomal miR-3198 in maternal plasma may indicate an endothelial functional disorder of trophoblasts. The study offered the in vitro evidence to properly understand the decrease of circulatory exosomal miR-3198 or the increase of placental WNT3 as a warning effect of PE. Besides, miR-3198/WNT3 targeting could be a possible therapeutic approach for the treatment of PE. The decrease of maternal circulatory exosome miR-3198 could be a valuable molecular marker for the prediction of preeclampsia.

However, our study has some limitations. First, The acquisition of samples occurred after the diagnosis of PE. The causal relationship between the decrease of exosomal miR-3198 in circulation and the occurrence of PE is unclear, and prospective studies are needed to clarify the relationship between the two. Second, the regulatory target genes of miR-3198 are not unique, and we only selected WNT3 as the downstream target gene for exploration. Future studies should explore more potential downstream target genes of miR-3198 to deepen our understanding of the role of miR-3198 in PE development. Finally, the current finds need to be further verified in suitable animal models in the future.

Our finds may provide new indicators for preeclampsia screening, thus allowing for early risk warnings and screening, which are of great importance for rapid preeclampsia management and intervention. Taken together, our results provide novel insights into maternal plasma detection for placental origin diseases and into novel exosomal mechanisms of PE development.

## Conclusion

This research opens a new avenue for clinical diagnosis and identification of therapeutic targets in PE. Maternal plasma exosomes and their cargo reflect the placental state and may serve as early markers of PE. Exosomal miRNAs can be used for screening and diagnosing PE.

## Data Availability

The data that support the findings of this study are available from the corresponding authors upon reasonable request.
